# Prognostic value of systemic immune-inflammation index in patients with metastatic renal cell carcinoma treated with systemic therapy: a meta-analysis

**DOI:** 10.3389/fonc.2024.1404753

**Published:** 2024-06-19

**Authors:** Juan Xu, Pingrun Chen, Shangqi Cao, Xu Hu, Xiang Li

**Affiliations:** ^1^ Operating Room, West China Hospital, Sichuan University/West China School of Nursing, Sichuan University, Chengdu, China; ^2^ Department of Gastroenterology and Hepatology, West China Hospital, West China Medical School, Sichuan University, Chengdu, China; ^3^ Department of Urology, West China Hospital, West China Medical School, Sichuan University, Chengdu, China

**Keywords:** prognostic value, systemic immune-inflammation index, metastatic renal cell carcinoma, systemic therapy, meta-analysis

## Abstract

**Objective:**

A novel systemic immune-inflammation index (SII), based on the neutrophils, lymphocytes, and platelet counts, is associated with the prognosis of several cancers, including non-metastatic renal cell carcinoma (RCC). In the present study, we evaluate the prognostic significance of SII in patients with metastatic RCC (mRCC) treated with systemic therapy.

**Method:**

Relevant studies were searched comprehensively from Web of Science, PubMed, Embase and the Cochrane Library up to January 2024. The pooled hazard ratio (HR) and 95% confidence interval (CI) were extracted from each study to evaluate the prognostic value of SII in patients with mRCC treated with tyrosine kinase inhibitor (TKI) or immune checkpoint inhibitor (ICI).

**Results:**

A total of 12 studies including 4,238 patients were included in the final analysis. High SII was significantly correlated to poor overall survival (OS, HR = 1.88; 95% CI 1.60–2.21; *P* < 0.001) and progression-free survival (PFS, HR = 1.66; 95% CI 1.39–1.99; *P* < 0.001). Stratified by therapy, high SII was also related to the poor OS (TKI: HR = 1.63, *P* < 0.001; ICI: HR = 2.27, *P* < 0.001) and PFS (TKI: HR = 1.67, *P* < 0.001; ICI: HR = 1.88, *P* = 0.002).

**Conclusion:**

In conclusion, high SII could serve as an unfavorable factor in patients with mRCC treated with systemic therapy. Stratified by therapies, the elevated SII was also associated with worse prognosis. Whereas, more prospective and large-scale studies are warranted to validate our findings.

**Systematic review registration:**

https://www.crd.york.ac.uk/prospero/display_record.php?ID=CRD42024522831, identifier CRD42024522831.

## Introduction

Renal cell carcinoma (RCC) is one of the common urological cancers, accounts for approximately 2% of all malignancies, with an estimated 431,288 new cases and 179,368 deaths in 2020 worldwide (1). Although most patients are localized disease and could undergo surgical resection with curative intent, about one-third of patients will develop metastatic disease recurrence (2, 3). Furthermore, 30% of patients present regional or distant metastases at initial diagnosis (2). Over the last decades, advancements in the treatment of metastatic RCC (mRCC) improved patients’ prognosis dramatically, such as the tyrosine kinase inhibitors (TKI) and immune checkpoint inhibitors (ICIs) (4, 5).

With the progress of the management of mRCC, identification of predictive markers would be of great value to patients’ treatment and long-term outcomes. The International metastatic renal cell carcinoma Database Consortium (IMDC) risk model is widely used for mRCC patients’ stratification and treatment selection. Recently, other potential biomarkers have been investigated for their prognostic and predictive value, including programmed cell death ligand 1 (PD-L1) expression, tumor mutational burden (TMB), molecular and genomic signatures, and clinical factors (6).

Evidences suggested that host inflammation response plays an important role in cancer progression by enhancing tumor angiogenesis and metastasis (7, 8). Peripheral blood parameters might reflect the cancer-related inflammatory phenomena. Previous reported have reported that prognostic value of neutrophil-lymphocyte ratio (NLR), platelet-lymphocyte ratio (PLR), prognostic nutrition index (PNI), and systemic immune-inflammation index (SII) in many cancers (9–12).

The SII is defined as follows platelet count × neutrophil count/lymphocyte count and has been found to be associated with the prognosis of several cancers, such as urothelial carcinoma, hepatocellular carcinoma, and non-metastatic RCC (12–14). The prognostic value of SII in mRCC patients treated with systemic therapy remains unclear. Therefore, we summarized all relevant studies and investigated the prognostic significance of SII in mRCC.

## Materials and methods

### Search strategy

The present study was performed based on the Preferred Reporting Items for Systematic Reviews and Meta-Analyses (PRISMA) Statement (15). Moreover, present study has been registered in PROSPERO (CRD42024522831). We comprehensively searched Embase, Web of Science, PubMed, and the Cochrane Library up to January 2024. Two independent reviewers performed the study search based on the search strategy (SII, systemic inflammation index, systemic immune-inflammation index) and (kidney cancer, renal cancer). We also screened the references of eligible studies to avoid the omission.

### Inclusion and exclusion criteria

Studies finally enrolled in the present study should meet the following criteria: (1) population-based studies, (2) involved patients with mRCC, (3) patients were treated with TKI or ICI, (4) SII was defined accurately and calculated based on the formula, (5) evaluate the prognostic value of SII, (6) available data such as hazard ratio (HR) and 95% confidence interval (CI) could be extracted. The following studies were excluded: (1) did not involve SII, (2) did not evaluate the prognostic value of SII, (3) insufficient data for HR and 95% CI, and (4) patients weren’t treated with systemic therapy. For the same cohort patients, we included the study with the largest and newest data.

### Data extraction and quality assessment

Two reviewers extracted the relevant data from eligible studies independently based on the predefined items: publication year, participant, study design, disease, therapy, number and ages of patients, the cutoff value of SII, clinical outcomes, and duration of follow-up. The Newcastle-Ottawa Quality Assessment Scale (NOS) was used to evaluate the quality of included studies, incorporating three main aspects: selection, comparability, and exposure/outcome. Total scores ranged from 0 to 9, a score of no less than 7 was considered as high quality.

### Statistical analysis

STATA (version 12, StataCorp, College Station, TX, USA) was applied to conduct all statistical analyses. We extracted and pooled HRs and 95% CIs through the inverse-variance method to investigated the prognostic value of SII in patients with mRCC. A random-effects approach was chosen over a fixed-effects approach, because using random effects is often preferred when performing a meta-analysis to guide patient treatment decision (16, 17). For the evaluation of heterogeneity across studies, the Cochran’s Q test and the Higgins’*I* ^2^ statistic were calculated. If the *I* ^2^ ≥ 50% or *P* < 0.10, the between-study heterogeneity was considered as significant (18). The sensitivity analyses were conducted to validate the stability of the final results by omitting each study in sequence. We also performed subgroup analysis and meta-regression to explore the potential source of heterogeneity. A two-sided *P*-value of < 0.05 was considered significant.

## Results

**Figure 1:**
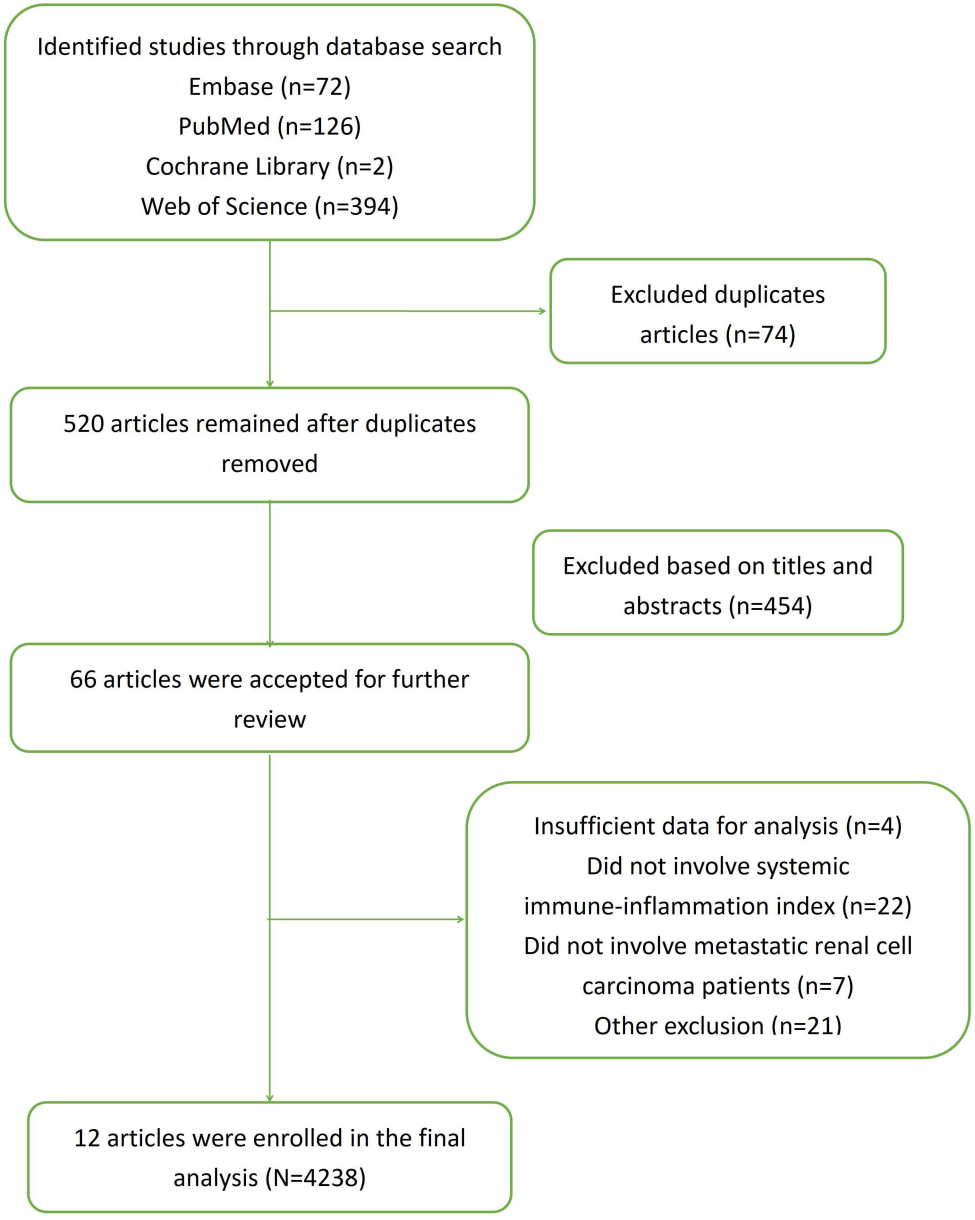
Flowchart of the literature search.

At first, 594 articles were identified based on the electronic database search. After excluding the 74 duplicated articles, the remaining 520 records were screened. According to the titles and abstracts, 66 studies were further reviewed detailedly. At last, a total of 12 studies incorporating 4,238 patients were included in the final analysis (19–30). The detailed information was illustrated in **Figure 1** (**Supplementary Table S1**).

### Clinical characteristic of the enrolled studies

Most studies were retrospective, while two studies were prospective (19, 28). Eight of the studies were multicenter. Half of studies included mRCC patients treated with ICIs, and the other six studies involved mRCC patients treated with TKIs. Most studies have a quite large sample size, ranging from 49 to 1,034. SII was calculated based on the formula (platelet×neutrophil/lymphocyte). The cutoff value of SII in each study is not consistent. All studies reported the overall survival (OS), seven studies reported progression-free survival (PFS). All studies were regarded as high quality (**Supplementary Table S2**). The detailed information was summarized in **Table 1**.

**Table 1:** Clinical characteristics of enrolled studies.

Study	Enrollment date/location	Study type	Intervention	Outcome	Number of patients	Age (years) Median (range)	Cutoff of SII (×10^9^/L)	Newcastle–Ottawa Quality Assessment Scale
Monteiro 2024 (30)	1 January 2017 to 1 February 2023/56 centers	Retrospective	First-line immune combinations	Overall survival Progression-free survival	1,034	64 (25–88)	1265	8
Anpalakhan 2023 (19)	October 2019 to January 2020/63 centers	Prospective	Immune checkpoint inhibitor	Overall survival	200	Not report	831	7
Korkmaz 2023 (20)	January 2015 to December 2021/Turkey/single center	Retrospective	First-line tyrosine kinase inhibitor	Overall survival Progression-free survival	110	Not report	782.56	7
Li 2022 (21)	1 June 2018 to 30 June 2022/China/single center	Retrospective	Immune checkpoint inhibitor	Overall survival Progression-free survival	52	56 (27–74)	1388.73	7
Stuhler 2022 (22)	As of May 2018/Germany/single center	Retrospective	First-line ipilimumab +nivolumab	Overall survival Progression-free survival	49	64.6 (39.9–83.5)	788	7
Yucel 2022 (23)	January 2007 to June 2020/Turkey/13 centers	Retrospective	First-line tyrosine kinase inhibitor	Overall survival Progression-free survival	706	Median (interquartile range) 60 (53–67)	756	7
Bugdayci Basal 2021 (24)	January 2012 to December 2019/Turkey/single center	Retrospective	First-line tyrosine kinase inhibitor	Overall survival	187	61 (34–86)	730	7
Rebuzzi 2021 (25)	October 2015 to November 2019/Italy/multicenter	Retrospective	Nivolumab	Overall survival Progression-free survival	571	61 (49–73)	720	8
Teishima 2020 (26)	January 2008 to January 2018/Japan/multicenter	Retrospective	First-line tyrosine kinase inhibitor	Overall survival	179	Median 65	730	8
Chrom 2019 (27)	2008–2016/Poland/two centers	Retrospective	First-line tyrosine kinase inhibitor	Overall survival	502	62 (22–88)	730	8
De Giorgi 2019 (28)	July 2015 to April 2016/Italy/multicenter	Prospective	Nivolumab	Overall survival	313	65 (40–84)	1375	8
Lolli 2016 (29)	January 2006 to December 2014/Italy/seven centers	Retrospective	First-line sunitinib	Overall survival Progression-free survival	335	63 (27–88)	730	7

### Overall survival

**Figure 2:**
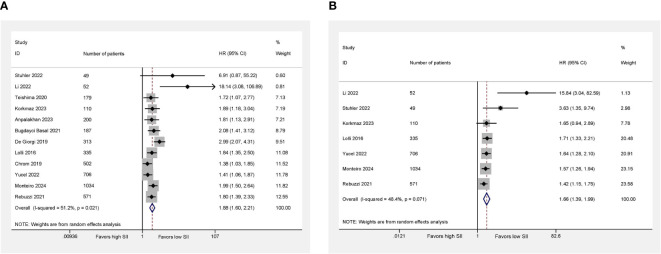
Higher SII was associated with worse OS **(A)** and PFS **(B)** in mRCC patients treated with systemic therapies. Right side (HR > 1) favors low SII, left side (HR < 1) favors high SII.

All studies including 4,238 patients reported OS. We observed moderate heterogeneity among studies, so the random effect was applied (*I* ^2^ = 51.2%; *P* = 0.021). A higher SII was significantly related to the worse OS compared with lower SII (HR = 1.88; 95% CI 1.60–2.21; *P* < 0.001; **Figure 2A**).

### Progression-free survival

Regarding PFS, seven studies involving 2,857 patients revealed relevant data. We also observed the evidence of heterogeneity (*I* ^2^ = 48.4%; *P* = 0.071). Using the random-effect model, we found that the patients with higher SII had a worse PFS compared with the patients with lower SII (HR = 1.66; 95% CI 1.39–1.99; *P* < 0.001; **Figure 2B**).

### Sensitivity analysis

**Figure 3:**
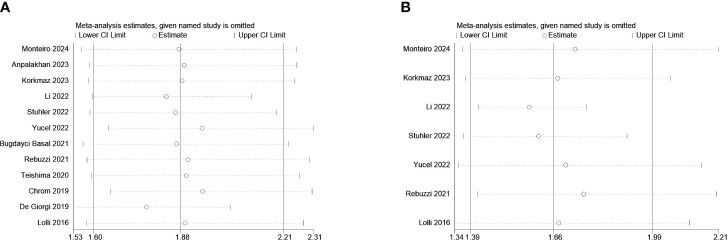
Sensitivity analysis for OS **(A)** and PFS **(B)**.

The sensitivity analysis for OS and PFS was conducted by eliminating each study to reflect the impact of the individual to overall. Consistently, we observed that removing any single study would not dramatically alter the trend of our results (**Figure 3**).

### Subgroup analysis and meta-regression

We performed subgroup analysis stratified by number of patients, study type, therapy, and enrollment. For the different types of studies, we both observed that high SII was significantly associated with poor OS. In the subgroup with large than 300 patients, high SII was also associated with the inferior OS and PFS, and in the <300 patients, we also detected the prognostic significance of SII. As for therapy, high SII was an unfavorable factor in patients treated with ICI or TKI. In addition, in studies from multicenter or single center, high SII was significantly related to the poor OS and PFS. Meta-regression revealed that none of these variables are significantly associated with the heterogeneity. The detailed information was summarized in **Table 2**.

**Table 2:** Subgroup analyses of overall survival and progression-free survival.

Subgroup	Variable	Number of studies	Model	HR (95% CI) *P*-value	*I* ^2^	*P*-value of meta-regression
Overall survival	All	12	Random	1.88 (1.60–2.21) < 0.001	51.2%	
Type	Prospective	2	Random	2.38 (1.46–3.89) 0.001	63.0%	0.148
	Retrospective	10	Random	1.77 (1.51–2.07) < 0.001	39.5%	
No. of patients	< 300	6	Random	2.06 (1.51–2.80) < 0.001	37.1%	0.573
	> 300	6	Random	1.81 (1.48–2.21) < 0.001	63.8%	
Therapy	Immune checkpoint inhibitor	6	Random	2.27 (1.68–3.06) < 0.001	59.7%	0.121
	Tyrosine kinase inhibitor	6	Random	1.63 (1.41–1.88) < 0.001	0	
Enrollment	Single center	4	Random	2.68 (1.47–4.88) 0.001	57.6%	0.355
	Multicenter	8	Random	1.80 (1.53–2.12) < 0.001	49.5%	
Progression-free survival	All	7	Random	1.66 (1.39–1.99) < 0.001	48.4%	
No. of patients	< 300	3	Random	3.65 (1.21–11.05) 0.022	73.0%	0.264
	> 300	4	Random	1.57 (1.40–1.76) < 0.001	0	
Therapy	Tyrosine kinase inhibitor	3	Random	1.67 (1.41–1.98) < 0.001	0	0.680
	Immune checkpoint inhibitor	4	Random	1.88 (1.26–2.80) 0.002	73.1%	
Enrollment	Single center	3	Random	3.65 (1.21–11.05) 0.022	73.0%	0.264
	Multicenter	4	Random	1.57 (1.40–1.76) < 0.001	0	

## Discussion

In the present study, we evaluate the association between SII and mRCC patients, observing that high SII was associated with the poor prognosis of mRCC. When stratified by therapies, high SII also predicts an inferior OS and PFS in mRCC patients treated with TKI or ICI. Moreover, we performed subgroup analysis and found all subgroup results are consistent with overall results. We only included the studies that provided the largest and newest data, which may affect the totality of data. However, this would likely be non-differential given the prognostic nature of the studies.

Local recurrence or metastasis is highly likely to occur in the RCC, nearly 30% of patients will develop local or distant recurrence after surgery (3). Meanwhile, one-third of patients suffer from metastasis initially (2). Although systemic therapy achieved great improvement of mRCC patients’ survival, not all patients could respond to these therapies. Many efforts to explore predictive markers are continuing.

The association between inflammation and malignancy has been widely explored in the past decades. Lots of studies have revealed an immunogenic nature of RCC (31). This immunogenic microenvironment may explain the antitumor efficacy of immune-related therapy used in mRCC treatment. While, the tumor infiltrating cells and their secretions may play a role in tumorigenesis, progression and clinical outcomes (32). It has been revealed that some peripheral markers of inflammation, NLR, PLR, and CRP were associated with prognosis of mRCC patients (33). Yucel et al. collected 706 mRCC patients treated with first-line TKI from multicenter and observed that pre-treatment high SII was considered a predictor of poor OS (HR = 1.39; *P* = 0.01) and PFS (HR = 1.60; *P* < 0.001) (23). In addition, SII might provide the similar predictive value as the IMDC and MSKCC, with the similar C-index values for OS and PFS in SII, IMDC, and MSKCC risk scores (23). Bugdayci Basal et al. demonstrated that in different IMDC risk groups, the patients with higher SII had a significantly worse OS compared with those with lower SII. And the SII may increase the predictive value of IMDC risk model in mRCC patients treated with TKI (24). Chrom et al. demonstrated that the addition of the SII to the IMDC model in place of neutrophil and platelet counts increased the model’s prognostic performance (27). Moreover, for mRCC patients received ipilimumab plus nivolumab in the first-line setting, high SII was also an unfavorable factor for OS and PFS (22). A prospective cohort of patients with mRCC treated with nivolumab also revealed that SII independently predicted OS (HR = 2.99; *P* < 0.001) (28). Recently, a retrospective study of 1,034 mRCC patients from 56 centers also revealed that a high SII is associated with poor oncological outcomes in patients treated with first-line immune combinations therapy (30). Wang et al. performed a meta-analysis and explored the prognostic value of SII in cancer patients receiving ICI. They found SII could predictive OS and PFS irrespective the cancer type, ICIs type and cutoff value of SII (34). Based on the abovementioned evidence, high SII could be served as an unfavorable factor in mRCC patients treated with systemic therapies. However, more large-scale studies are required to verify our findings.

The potential mechanism for the prognostic significance of this combination might be explained by the functions of neutrophil, platelet, and lymphocyte. Neutrophils can promote cancer development through directly interacting with tumor cells. Neutrophils can secrete proinflammatory cytokine and chemokine related to the remodeling of the tumor microenvironment and have a tumor-promoting effect (35). Platelets have been reported to regulate tumor angiogenesis, protect tumor cells from cytolysis, and contribute to tumor metastasis (36). As a major cellular immunity component in humans, lymphocytes are implicated in killing the host cancer cells by cell-mediated immunization. Therefore, the decreased lymphocytes may cause a weak anti-tumor activity and lead to cancer progression (24).

SII had significance in clinical practice. SII was calculated based on the neutrophil, platelet, and lymphocyte, which is convenient, easily obtained and commonly tested before the treatment. SII could predict the prognosis of patients, which could be used for mRCC patients’ managements. However, the individual conditions should be considered during the treatment strategy decision.

Our study is not devoid of shortcomings. First of all, total 12 studies consisting of 4,238 patients were included, which is not a relatively large sample and may limit the power of final results. Next, almost all studies were retrospective studies with the potential inherent bias, resulting heterogeneity. Therefore, we conducted sensitivity analysis and subgroup analysis. We only included the studies that provided the largest and newest data, which may affect the totality of data. But this would likely be non-differential given the prognostic nature of the studies. At last, although we performed subgroup analyses and meta-regression, there are several factors that are not available and may result heterogeneity such as detailed treatments and comorbidity, so we could not conduct additional analyses.

## Conclusion

In conclusion, high SII could serve as an unfavorable factor in patients with mRCC treated with systemic therapy. Stratified by therapies, the elevated SII was also associated with worse prognosis. Whereas, more prospective and large-scale studies are warranted to validate our findings.

## Data availability statement

The original contributions presented in the study are included in the article/**Supplementary Material**. Further inquiries can be directed to the corresponding author.

## Author contributions

JX: Writing – original draft, Methodology, Formal analysis, Data curation. PC: Writing – original draft, Methodology, Formal analysis, Data curation. SC: Writing – original draft, Methodology, Formal analysis, Data curation. XH: Writing – original draft, Data curation. XL: Writing – review & editing, Supervision, Investigation, Conceptualization.
