# Supplementary material for: Prognostic value of systemic immune-inflammation index in patients with metastatic renal cell carcinoma treated with systemic therapy: a meta-analysis
Source: Front Oncol. 2024 Jun 19;14:1404753. doi: 10.3389/fonc.2024.1404753 (PMC11220114; doi:10.3389/fonc.2024.1404753)
Supplement: Supplementary file 1 [file Table_1.docx]

Supplementary Table 1. Search terms.

| Data Base | Detailed search terms |
| --- | --- |
| PubMed | ((kidney cancer) OR (renal cancer)) AND (((SII) OR (systemic inflammation index)) OR (systemic immune-inflammation index)) |
| Embase | 1 renal cancer.mp. or exp kidney cancer/  2 kidney cancer.mp. or exp kidney cancer/  3 SII.mp.  4 systemic inflammation index.mp.  5 systemic immune-inflammation index.mp.  6 1 or 2  7 3 or 4 or 5  8 6 and 7 |
| Web of Science | 1. (TS=(renal cancer)) OR TS=(kidney cancer)  2. ((TS=(systemic immune-inflammation index)) OR TS=(systemic inflammation index)) OR TS=(SII)  3. #1 AND #2 |
| Cochrane Library | 1 systemic immune-inflammation index.mp. (38)  2 SII.mp. (171)  3 renal cancer.mp. or exp Kidney Neoplasms/ (1875)  4 kidney cancer.mp. or exp Kidney Neoplasms/ (2128)  5 1 or 2 (179)  6 3 or 4 (2254)  7 5 and 6 (2) |

Supplementary Table 2. Newcastle-Ottawa Scale for assessing the quality of studies in meta-analysis

| Study | Selection | | | | Comparability | Outcome | | | Quality score |
| --- | --- | --- | --- | --- | --- | --- | --- | --- | --- |
|  | Representativeness of the Exposed Cohort | Selection of the Non-Exposed Cohort | Ascertainment of Exposure | Demonstration That Outcome of Interest Was Not Present at Start of Study | Comparability of Cohorts on the Basis of the Design or Analysis | Assessment of Outcome | Was follow-up long enough for outcomes to occur | Adequacy of follow up of cohorts |  |
| Monteiro 2024 | * | * | * | * | * | * | * | * | 8 |
| Anpalakhan 2023 | * | * | * |  | * | * | * | * | 7 |
| Korkmaz 2023 | * | * | * |  | * | * | * | * | 7 |
| Li 2022 | * | * | * |  | * | * | * | * | 7 |
| Stuhler 2022 | * | * | * |  | * | * | * | * | 7 |
| Yucel 2022 | * | * | * |  | * | * | * | * | 7 |
| Bugdayci Basal 2021 | * | * | * |  | * | * | * | * | 7 |
| Rebuzzi 2021 | * | * | * | * | * | * | * | * | 8 |
| Teishima 2020 | * | * | * | * | * | * | * | * | 8 |
| Chrom 2019 | * | * | * | * | * | * | * | * | 8 |
| De Giorgi 2019 | * | * | * | * | * | * | * | * | 8 |
| Lolli 2016 | * | * | * |  | * | * | * | * | 7 |
